# Risk perception of COVID-19, depressive symptoms and Internet addiction among undergraduates: a longitudinal study

**DOI:** 10.3389/fpubh.2024.1487472

**Published:** 2025-01-23

**Authors:** Hongpo Zhang, Dandan Zhang, Wanghua Ji, Shun Peng

**Affiliations:** ^1^School of Management, Henan University of Chinese Medicine, Zhengzhou, China; ^2^Mental Health Education and Counseling Center, Henan University of Chinese Medicine, Zhengzhou, China; ^3^School of Education, Huanggang Normal University, Huanggang, China

**Keywords:** risk perception, COVID-19, depressive symptoms, Internet addiction, post-traumatic growth

## Abstract

**Background:**

The COVID-19 pandemic has caused serious negative psychological effects worldwide, relatively little research has been performed on the potential enduring effects of COVID-19 on people’s emotional health and Internet addiction. This study was to examine the longitudinal associations between risk perception of COVID-19, depressive symptoms, and Internet addiction among Chinese undergraduates.

**Methods:**

We conducted a two-wave longitudinal survey by convenience sampling, a total of 1,153 Chinese undergraduate students completed questionnaires measuring their COVID-19 risk perception and post-traumatic growth (PTG) in December 2022 (T1) via an online survey. Subsequently, 1,008 of the T1 participants (87.42%) completed the depressive symptoms scale and Internet addiction scale 6 months later, in June 2023 (T2).

**Results:**

(1) Risk perception of COVID-19 was significantly and positively predicted depressive symptoms and Internet addiction 6 months later; (2) Depressive symptoms played a mediating role between risk perceptions and Internet addiction; (3) PTG moderated the first-stage link between risk perception and Internet addiction, and this relationship was more robust for low PTG than for high PTG.

**Conclusion:**

These findings advance our understanding of the relationship and mechanisms between risk perception of COVID-19 and Internet addiction, and further support developing interventions to strengthen PTG for mitigating negative outcomes during major crises.

## Introduction

As a significant global public health crisis, the COVID-19 pandemic has profound and long-term impacts on individuals’ mental health, eating habits and physical activity (1, 2). During the pandemic, home quarantines, lockdowns, and limited interpersonal contact were advocated and enforced globally, which prevented students from attending school (3) and athletes from training regularly (4, 5). Internet has become the primary means by which citizens could access dynamic information, maintain social connections, and learn (6, 7). However, several studies have confirmed that the prevalence of Internet addiction increased significantly during the COVID-19 pandemic (8, 9).

The COVID-19 pandemic is characterized by clear “human-to-human” transmission and high uncertainty and uncontrollability, posing a major health threat to human survival (10). According to the stress and coping theory, stressful events affect an individual’s response by influencing their cognitive appraisal and coping strategy tendencies (11, 12). Risk perception is the subjective awareness and judgment of various external objective risks (13, 14), which may influences an individual’s affective response (15) and self-protective behavior (16). Studies have found that those with higher risk perceptions tend to overestimate the severity of diseases (17) and experience higher levels of emotional distress (18). In addition, Individuals with higher perceived severity of the pandemic were more likely to search for health-related information on the Internet and to be at higher risk for Internet addiction (19).

Although the COVID-19 pandemic is under control and people have returned to normal life, its impact on citizens’ mental health and behavior may continue (5). Since the resumption of normal activities on December 27, 2022 following the end of lockdown in China (20), sporadic cases have continued to emerge in some areas, and many people have experienced second or even multiple infections. Therefore, it is essential to explore the enduring effects and psychological mechanisms of COVID-19 risk perception on Internet addiction.

### Depressive symptoms as a mediator

The outbreak of COVID-19 pandemic has a significant impact on individuals’ emotional and mental health. A meta-analysis of the eighty-nine studies (*n* = 1,441,828) showed that the pooled prevalence of depressive symptoms, anxiety symptoms, and sleep disturbances during the COVID-19 pandemic was 34, 32 and 33%, respectively (21). The rapid spread and highly contagious nature of the COVID-19 pandemic may raise fears of unobservable hazard (18). When people perceive risks that cannot be avoided (e.g., natural catastrophes, pandemics), they may experience a sense of helplessness, frustration, and depression (22–24). Previous studies have found a positive association between risk perceptions of COVID-19 and depressive symptoms (25).

Long-term exposure to fear and depression is undoubtedly distressing, and individuals may seek ways to escape from unpleasant feelings. The Internet can provide an important means of relieving negative emotions and entertaining individuals, especially during the COVID-19 pandemic when mobility was limited. According to the Interaction of Person-Affect-Cognition-Execution (I-PACE) model, individuals may turn to the Internet to cope with difficulties in their lives and seek compensation from the Internet (26, 27). Studies have demonstrated that people experiencing negative emotions are more inclined to surf the Internet or play online games (28, 29); moreover, systematic reviews have revealed that depressive symptoms are significant contributors to the prevalence of increased Internet addiction during the pandemic (8). Thus, depressive symptoms may mediate the association between risk perception during the pandemic and Internet addiction.

### Post-traumatic growth as a moderator

Experiencing or witnessing a traumatic episode may result in a range of mental health problems (30, 31). Yet, traumatic events can also prompt positive changes like post-traumatic growth (PTG). PTG refers to the positive psychological changes an individual experiences after a traumatic event, including engaging in self-awareness, reflecting on relationships with others, and thinking about the meaning of life (32). PTG may occur in populations that have experienced different traumatic events (31, 33). Moreover, reports of PTG were common during the pandemic, with several studies documenting relatively high levels of PTG among samples worldwide (34, 35).

According to the stress-buffering hypothesis, specific coping strategies can alleviate the negative impacts of stress (36, 37). PTG often serves as an effective coping strategy for managing traumatic experiences and reduces the negative impact of individuals experiencing past traumatic or stressful events (38, 39). Many people report positive transformative changes when describing their experiences of PTG (40, 41); these include recognizing one’s psychological strength (34), identifying as a survivor (42), and building closer connections with others (43). Studies have demonstrated that PTG moderates the relationship between post-traumatic stress and well-being (39, 44). Furthermore, PTG attenuates the association between risk perception of cancer and psychosocial adjustment (36, 37, 45). Thus, PTG may moderate the relationship between risk perception of COVID-19 and Internet addiction.

### The present study

This study aims to extend the existing literature by examining the longitudinal associations between risk perception of COVID-19, depressive symptoms, and Internet addiction among Chinese undergraduates. Three hypotheses were proposed: (H1) Risk perception is positively correlated with Internet addiction; (H2) Depressive symptoms mediate the association between risk perception and Internet addiction; and (H3) PTG moderates the relationship between risk perception and Internet addiction.

## Methods

### Participants

The study conducted two waves of longitudinal surveys through convenience sampling. Data were collected at two time points (T1, T2) during potential participants’ first year of college in Zhengzhou, China. We gathered the T1 data via an online survey administered to 1,153 students in December 2022. T2 data were collected 6 months later, in June 2023, and 1,008 of the T1 participants (87.42%) responded at T2. The inclusion criteria for participants in this study include: signed informed consent, completed two surveys, and no missing or abnormal values in personal information and research variables. The data that did not meet the above criteria were excluded. Among the final sample, 384 participants were male (38.10%) and 624 were female (61.90%). Their ages ranged from 16 to 21 (*M* = 18.29, SD = 0.70). Risk perception and PTG were measured at T1; Internet addiction and depressive symptoms were measured at T2. The participants were fully informed about the study and provided informed consent online prior to data collection.

### Measurements

#### Risk perception of COVID-19

Risk perception was assessed with the COVID-19 Risk Perception Scale (46). This scale contains 9 items (e.g., “I am highly susceptible to COVID-19”) covering three dimensions of epidemics: susceptibility, severity, and controllability. Items were rated on a 5-point Likert scale ranging from 1 (*totally disagree*) to 5 (*totally agree*). The scale has good validity and reliability (23). Cronbach’s *α* was 0.76 in this study.

#### Depressive symptoms

Depressive symptoms were assessed with the Depression Anxiety Stress Scale-21 (DASS-21) (47). The scale comprises three subscales: depression, anxiety, and stress. Depressive symptoms was measured with seven items (e.g., “I felt life was meaningless”); each item was rated on a 4-point Likert scale ranging from 0 (*does not apply to me*) to 3 (*applies to me very much or most of the time*). Based on criteria for the depression subscale of the DASS-21 manual, after multiplying the scale scores by two, the rating scale for each category was *normal* (0–9 points), *mild* (10–13 points), *moderate* (14–20 points), *severe* (21–27 points), or *very severe* (≥28 points). This scale has good validity and reliability (48, 49). Cronbach’s *α* was 0.91 in this study.

#### Internet addiction

Internet addiction was assessed by the Young’s Internet Addiction Diagnostic Inventory (50). The inventory included eight items (e.g., “Do you use the Internet as a way to escape from problems and deal with negative emotions?”). Participants responded dichotomously (*yes* or *no*) to each item, and those who answered *“yes”* to six or more items were categorized as having Internet addiction tendencies. According to a previous study (29), Internet addiction was considered a continuous variable, with the mean of all items serving as an indicator of Internet addiction. Cronbach’s *α* was 0.83 in this study.

#### Post-traumatic growth

PTG was assessed by the Post-Traumatic Growth Inventory-Revised (31, 51). The inventory contains 22 items in three subscales: perceived changes in self, a changed sense of relationship with others, and a changed philosophy of life. Each item was rated on a 5-point Likert scale ranging from 0 (*no change*) to 5 (*very high degree of change*). This scale has good validity and reliability (52). Cronbach’s *α* was 0.95 in this study.

### Data processing

SPSS 19.0 was used to conduct descriptive and correlation analyses, and PROCESS was used for moderated mediation analyses. In the path analyses, all variables were standardized, and 5,000 resamples were used to obtain confidence intervals (CIs) by bootstrapping.

## Results

### Common method bias

We used Harman’s single-factor test to assess the extent of common method bias (53). The analysis revealed that the initial eigenvalues of eight factors were greater than 1, and a single factor explained 24.61% of the total variance (lower than 40%), indicating no serious common method bias issues.

### Descriptive statistics

The DASS-21 criteria indicate that 7.74% (78) of participants experienced mild depressive symptoms, 15.38% (155) experienced moderate depressive symptoms, and 7.34% (74) experienced severe or above depressive symptoms. Moreover, the prevalence of Internet addiction was 24.40% in this study.

Descriptive statistics and correlation analyses indicated that risk perception was positively related to depressive symptoms (*r =* 0.13, *p* < 0.001) and Internet addiction (*r =* 0.17, *p* < 0.001), and depressive symptoms were positively associated with Internet addiction (*r =* 0.37, *p* < 0.001) (Table 1).

**Table 1:** Descriptive statistics and correlations of the variables (*N* = 1,008).

Variables	M ± SD	Skewness	Kurtosis	Correlation			
				1	2	3	4
1.Risk perception (T1)	26.17 ± 5.33	−0.38	0.75	−			
2.Post-traumatic growth (T1)	82.48 ± 20.98	−0.78	0.56	0.10**	−		
3.Depressive symptoms (T2)	6.84 ± 7.97	1.39	1.75	0.13***	−0.02	−	
4.Internet addiction (T2)	2.74 ± 2.44	0.63	−0.68	0.17***	−0.05	0.37***	−

^**^p < 0.01, ^***^p < 0.001.

### The mediating effect of depressive symptoms

**Figure 1:**
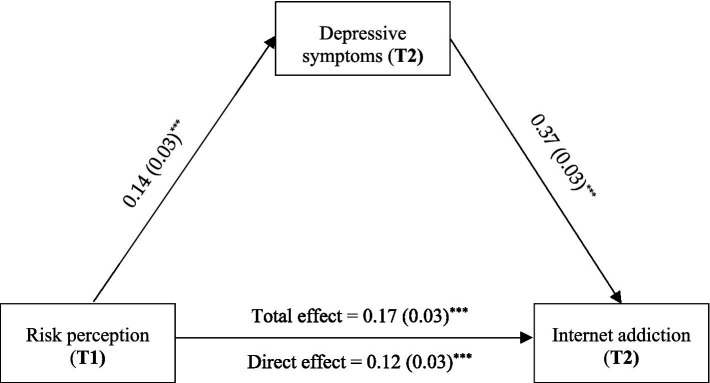
The mediation model of depressive symptoms between risk perception and Internet addiction (^***^p < 0.001).

To examine the mediating effect of depressive symptoms in the association between risk perception and Internet addiction, the PROCESS (Model 4) method was employed with age and gender controlled. As presented in Figure 1, risk perception positively predicted depressive symptoms (*β* = 0.14, *p* < 0.001), and depressive symptoms positively predicted Internet addiction (*β* = 0.37, *p* < 0.001). The direct relationship between risk perception and Internet addiction was significant (*β* = 0.12, *p* < 0.001). Hence, depressive symptoms partially mediated the association between risk perception and Internet addiction (*β* = 0.05, 95% CI [0.02, 0.08]). The mediating effect accounted for 29.41% of the total effect of risk perception on Internet addiction.

### The moderated mediation model

We used PROCESS (Model 59) to examine the moderating effect of PTG on the relationship between risk perception and Internet addiction. The interaction of risk perception and PTG on depressive symptoms was significant (*β* = −0.07, *p* < 0.01), indicating that PTG moderated the first-stage relationship between risk perception and Internet addiction.

**Figure 2:**
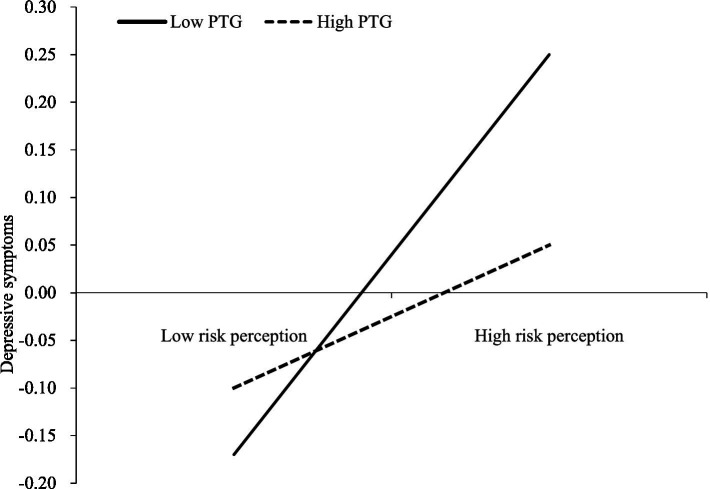
The relationship between risk perception and depressive symptoms at two PTG levels. PTG, post-traumatic growth.

To elaborate the interaction effect, we conducted a simple slope analysis. Figure 2 illustrates the relationship between risk perception and depressive symptoms at two levels of PTG (M − 1SD and M + 1SD), and the effect of risk perception on depressive symptoms was more substantial for low PTG (*β* = 0.09, 95% CI [0.04, 0.13]) than for high PTG (*β* = 0.02, 95% CI [−0.01, 0.66]).

## Discussion

The long-term impacts of the COVID-19 pandemic on mental health have been of significant concern (1, 5). This study explored the longitudinal relationship between risk perception of COVID-19 and Internet addiction among Chinese undergraduate students, as well as the mediating and moderating factors. These findings contribute to our understanding of the long-term effects of risk perception of COVID-19 on Internet addiction and provides further insights into potential intervention programs aimed at reducing Internet addiction.

In line with previous finding (19), this study demonstrated that risk perception of COVID-19 positively predicts Internet addiction 6 months later. As a highly infectious, pathogenic, and stressful event, the COVID-19 pandemic constitutes a major public health threat to individuals worldwide. Notably, the first survey took place during the initial period of full resumption of normal activities following the pandemic, which may contribute to the triggering of risk perceptions. Previous studies have found that individuals perceiving high risk of the pandemic are more likely to utilize the Internet to follow pandemic-related developments (8), interact through social media (17), and check for symptoms in themselves (54). Such Internet use behaviors are likely to increase the risk of developing an addiction to the Internet.

Furthermore, the study revealed that depressive symptoms played a mediating role in the relationship between risk perception and Internet addiction. These findings provide empirical evidence to support the I-PACE model (26, 27), suggesting that affective responses (depressive symptoms) serve as mechanism variables between perceived external stimuli and Internet addiction. The threat posed by the COIVD-19 pandemic is significant and beyond the control of individuals, leading to feelings of powerlessness and helplessness. These emotions may consequently result in depressive moods. The Internet thus has become an important means for individuals to fulfill their needs and alleviate their depression. Previous studies have noted a positive association between risk perception and depression (55), which in turn increases use of the Internet for stress relief (56).

As hypothesized, the results demonstrated that PTG moderated the first-stage relationship between risk perception and Internet addiction. Furthermore, the association between risk perception and depressive symptoms was more robust for individuals with low PTG than for those with high PTG. These results provide empirical support for the stress-buffering hypothesis (36, 37) and suggest that PTG serves as a coping mechanism that buffers the effect of risk perception on depressive symptoms and Internet addiction. PTG is characterized by positive changes experienced in the context of life crises; these include an increased sense of personal strength and wisdom, more meaningful interpersonal relationships, and new appreciation for each day (52, 57). People with high PTG beliefs have more optimism, possess higher self-efficacy, and perceive adversity as a growth opportunity to cope with traumatic experiences (58). Hence, people with high PTG beliefs may be more adept at managing their emotions effectively (31, 45), thereby reducing their reliance on the Internet (59). Therefore, interventions on enhancing PTG may have higher efficacy in reducing depressive symptoms and further mitigating the risk of Internet addiction.

Finally, the results of this study demonstrated that the prevalence of depressive symptoms and Internet addiction was 30.46 and 24.40%, respectively. These results are in accordance with previous studies on depressive symptoms and Internet addiction (8, 60). Meta-analysis results demonstrated that prior to the outbreak of the COVID-19 pandemic, the prevalence of depression was 23.8% among Chinese undergraduate students (61), while the prevalence of depressive symptoms up to 34% during the COVID-19 pandemic (21). Throughout the pandemic, the fear of the disease, lockdown situations, and high uncertainty about the future increased stress, anxiety, and depression levels globally (62). These findings provide further evidence that pandemics have long-lasting effects on individuals’ mental health (9), which has practical significance for university mental health services and broader public health institutions for ongoing monitoring and improving adolescent mental health in the aftermath of the COVID-19 pandemic (63).

Despite these findings, some limitations should be noted. First, we only collected two waves of longitudinal data over a period of 6 months, which may have prevented us from identifying stable associations between risk perception, depressive symptoms, and Internet addiction over time. Second, the homogeneity of the sample, which consisted entirely of participants from China, may limit the generalizability of our results. In particular, China’s strict lockdown policies and centralized controls during the COVID-19 pandemic outbreak may have amplified the psychological impact of risk perceptions. Future research could employ multiple methods and longer follow-up studies to examine the stability of the results in other cultural contexts.

## Conclusion

This study explored the longitudinal relationship between risk perception of COVID-19 and Internet addiction. We found that depressive symptoms mediated the link between risk perception and Internet addiction. Meanwhile, PTG buffers the effect of risk perception on depressive symptoms and Internet addiction. These findings contribute to deepen understanding of the long-term impact and mechanisms of COVID-19 risk perception on depressive symptoms and Internet addiction, and further support the design of intervention programs to strengthen PTG for mitigating negative consequences during major crises.

## Data Availability

The raw data supporting the conclusions of this article will be made available by the authors, without undue reservation.
